# Does the extent of neck surgery based on preoperative calcitonin level influence survival in medullary thyroid carcinoma: a retrospective tertiary centre experience

**DOI:** 10.1308/rcsann.2024.0033

**Published:** 2024-04-25

**Authors:** W Ansley, A Kamyab, L Noden, B Odutoye, P Williamson, KH Wong, P Dent, A Sharma, A Weller, G Pitiyage, E Ofo

**Affiliations:** ^1^ St George’s University Hospitals NHS Foundation Trust, UK; ^2^ St George's University of London, UK; ^3^ University of Cambridge, UK; ^4^ Royal Marsden NHS Foundation Trust, UK

**Keywords:** Medullary thyroid carcinoma, Calcitonin, Thyroid cancer, Neck dissection

## Abstract

**Introduction:**

Medullary thyroid carcinoma (MTC) is a rare thyroid cancer arising from the calcitonin-secreting parafollicular cells. Previous studies suggested a preoperative calcitonin level >200ng/l is an indication for prophylactic lateral neck dissection (LND) to remove micrometastases and improve survival outcomes.

**Methods:**

This retrospective single-centre study assessed the efficacy of preoperative calcitonin levels as a marker for determining need for prophylactic LND in MTC. Data were obtained on demographics, preoperative calcitonin levels, size and laterality of index tumour, type of neck dissection (central neck dissection (CND), LND), nodes removed, levels with involved nodes, number of nodes histologically involved, mortality, adjuvant therapy and locoregional recurrence.

**Results:**

A total of 33 patients were identified from St George's University Hospitals NHS Foundation Trust between 1 January 2001 and 19 August 2021; 8 were excluded for data quality issues. Of the 18 classified with a high preoperative calcitonin level (>200ng/l), 10 (56%) had a LND and 8 (44%) had a CND. In the low-calcitonin group, three (43%) patients had a CND only and four (57%) had a LND. There was no difference in absolute or disease-free survival between the low and high groups (*p*=0.960, *p*=0.817), or between those who had a CND and LND in the high-calcitonin group (*p*=0.607, hazard ratio (HR) 0.55; *p*=0.129, HR 8.78).

**Conclusion:**

There was no statistically significant difference in outcomes between high and low calcitonin groups. A selective approach to performing LND in MTC patients based on clinical and imaging findings suggesting disease presence in the lateral neck should be explored further.

## Introduction

Medullary thyroid carcinoma (MTC) is a rare cancer arising from the calcitonin-secreting parafollicular cells (C-cells) of the thyroid gland. Several studies have underlined the value of calcitonin as a tumour marker for MTC.^1^

MTC represents 1–2% of thyroid cancers, making it the third most common thyroid cancer.^2^ Of the total number of cases, 25% are hereditary, whereas 75% are sporadic.^3^ The hereditary form is part of the multiple endocrine neoplasia type 2 (MEN-2) syndromes, which are associated strongly with *RET* proto-oncogene mutation.^4^

Presentation differs between patients, potentially including a neck mass, dysphagia or dysphonia from compressive effects, or recurrent laryngeal nerve involvement. Vasomotor flushing and loose stools may result from coincident peptide secretion.^5^ Up to 80% of patients have regional lymph node metastases at presentation, with 20% having distant metastases. Despite this, the average five-year survival rate is 73–97%, with survival predictors including TNM stage, extent of disease at diagnosis and degree of surgical intervention.^6^

Following family history screening for MTC or other endocrinopathies, initial investigations include ultrasound (US), fine-needle aspiration cytology (FNAC) and biochemistry. A baseline calcitonin value may confirm the diagnosis and indicate the disease extent and likelihood of remission.^7–10^ Neck computed tomography (CT)/magnetic resonance imaging (MRI) is used to identify locally advanced disease.

Current guidelines use calcitonin levels to support diagnosis and guide the extent of surgical management. Basal calcitonin concentrations >60ng/l to 100ng/l are pathognomonic of MTC. There is greater diagnostic uncertainty at the lower levels of calcitonin.^11,12^

Typical MTC treatment involves total thyroidectomy and central neck dissection (CND) to the level of the innominate artery. This aims to achieve locoregional control in the neck and superior mediastinum.^5,13^ Neck dissection to remove cervical lymph nodes is necessary, as MTC cells are unresponsive to radioiodine and hormonal manipulation.^13^

In small, sporadic (RET-negative) MTC, following preoperative basal calcitonin assessment, most patients are offered total thyroidectomy and CND. Additionally, patients with clinically/radiologically involved lymph nodes in the lateral compartment should have a selective lateral neck dissection (LND). Those with obvious central node metastases should also be considered for ipsilateral prophylactic LND as the risk of lateral node involvement is 70%.^5,14^

The current guidelines rely heavily on calcitonin levels to guide the necessity of LND. The cut-off to undertake a prophylactic neck dissection has been cited as 200ng/l.^10,15^ In this study, we aim to evaluate the necessity of LND based on calcitonin levels by retrospectively reviewing postoperative pathology reports of this patient cohort.

## Methods

### Study population

The study population was obtained from St. George's Hospital pathology database using the master code: T96000 (THYROID) and secondary reporting code: M85103 (MEDULLARY CARCINOMA). The timeframe explored ran from 1 January 2000 to 19 August 2021. This timeframe was based on data availability in the online database. Patient data were retrieved manually from NHS systems including iCLIP and electronic patient record (EPR) using the patient’s individual medical record number (MRN). The data were reviewed for study inclusion and exclusion based on the criteria below and collated on Microsoft Excel, ensuring compliance with data protection policies and patient anonymisation.

### Inclusion and exclusion criteria

All patients identified using the codes above within the specified timeframe were initially included.

Patients were subsequently excluded for:

• missing preoperative calcitonin data on iCLIP and EPR;
• missing histology report details including nodes removed, nodes involved and TNM staging.

In cases of ambiguity, senior otorhinolaryngologists reviewed the patient’s records to determine inclusion or exclusion.

### Data collection

This was a retrospective study. Data were obtained on age, sex, calcitonin levels (pre- and postoperatively), RET mutation presence, index tumour size, laterality of index tumour, type of neck dissection (central, lateral), lymph node levels removed (1–6, left, right or central), levels with cancerous nodes, number of nodes removed, number of nodes histologically involved, presence of extracapsular extension, TNM stage, complications (bleeding, nerve injury and chyle leak), 30-day mortality, adjuvant therapy and locoregional recurrence. Furthermore, oncological survival data were collected. Patient MRN numbers were collected to enable later access for reference if needed.

The patient's lead clinician was recorded to enable discussion in the case of ambiguity in the patient notes. Clinical data nearest to the time of the operation were collected.

For patients with multiple preoperative calcitonin readings, the highest value was taken.

### Ethics

Ethical approval was not needed due to the study’s retrospective nature and use of routinely collected data.

### Statistical analysis

To mitigate detrimental effects on data quality, patient records were excluded if data were incomplete. Examples include the absence of preoperative calcitonin levels and lack of confirmation in the histopathology of the removed nodes.

The patients were split into two cohorts based on preoperative calcitonin level, with the high group defined as >200 ng/l. This cut-off was based on a 2010 study by Machens *et al*,^10^ who found that the likelihood of metastases to lateral neck nodes increased with preoperative calcitonin above 50, with lateral neck nodes on the contralateral side to the primary being involved more with a calcitonin >200ng/l. As such, they suggested that patients with preoperative calcitonin levels between 50 and 200ng/l would require an ipsilateral neck dissection, and those with a preoperative calcitonin >200ng/l would require a contralateral neck dissection (i.e. a lateral neck dissection contralateral to the side of the thyroid primary tumour) in addition to the ipsilateral dissection.

Correlation analysis was carried out to investigate the relationship between calcitonin levels and index tumour size. Independent *t*-tests were carried out to assess for differences in the number of nodes removed, nodes involved, and their proportion between the high- and low-calcitonin group. Overall and disease-free survival were calculated from the time of the operation to the latest follow-up or death using Kaplan–Meier methodologies.

Statistical analyses were carried out in R (version 4.2) or GraphPad Prism (version 8.0), depending on statistician preference. Significance was set at α=0.05, and all *p*-values were two-sided.

## Results

### Demographics and cancer characteritics

A total of 33 patients were identified from St George's University Hospitals NHS Foundation Trust. Eight patients were removed due to data-quality issues. The mean patient age was 50.8 years (range, 31–80); 10 patients (40%) were male and 15 (60%) were female. The median preoperative calcitonin level was 1,070ng/l (range, 2.2–41,080). Three patients (12%) had contralateral disease, with all being in the high-calcitonin group, and six (24%) had an identified RET mutation. A total of 22 patients had a total thyroidectomy, 2 a hemithyroidectomy and 1 a subtotal thyroidectomy. The decision to perform hemithyroidectomy was based on patient wishes in one case. The other case was a completion hemithyroidectomy. The patient who underwent a subtotal thyroidectomy had a T4 tumour; a total thyroidectomy was technically challenging, hence the procedure was recorded as subtotal.

Using 200ng/l as the preoperative calcitonin level cutoff, 18 patients were classified as high and the remaining 7 as low. Patient demographics are shown in Table 1.

**Table 1:** Patient demographics and cancer characteristics

	Total (*N*=25)	Low-calcitonin group (*N*=7)	High-calcitonin group (*N*=18)
Mean age, years (range)	50.8 (31–80)	60.4 (40–75)	47.0 (31–80)
Gender (male/female)	10/15	3/4	7/11
Median calcitonin level, ng/l (range)	1,070.0 (2.5–41,080)	17.0 (2.5–195)	3,985.0 (299–41,080)
RET mutation (yes/no)	6/19	2/5	4/14
Contralateral disease (yes/no)	3/22	0/7	3/15
CND/LND	11/14	3/4	8/10
Index tumour size, mm (range)	22.1 (5–50)	11.4 (5–21)	26.3 (8–50)
Preoperative nodal involvement (cN1) (yes/no)	11/14	4/3	7/11
Postoperative nodal involvement (pN1) (yes/no)	20/5	5/2	15/3

CND = central neck dissection; LND = lateral neck dissection.

The decision to undertake CND, ipsilateral LND or bilateral LND, as well as which levels were involved, was on a patient-by-patient basis, based on the multidisciplinary team (MDT) discussion. Factors including clinical examination findings, calcitonin levels, radiology and pathology results were accounted for.

Of the 18 patients in the high-calcitonin group, 10 (56%) had a LND with CND, and 8 (44%) had a CND alone. In the low-calcitonin group, three (43%) patients had a CND alone and four (57%) had a LND with CND.

In the high-calcitonin group, all ten (100%) patients who underwent a LND had positive cervical nodal disease. Nine of these (90%) had lateral neck node involvement. Two of the ten (20%) had an ipsilateral LND and eight (80%) had a bilateral LND. Both (100%) who had an ipsilateral LND and seven of the eight (87.5%) who underwent bilateral LND had lateral nodal involvement. In the low-calcitonin group, all four (100%) patients who underwent a LND had an ipsilateral lateral dissection. All four (100%) had positive cervical nodal disease, with three (75%) having lateral neck node involvement.

Clinically evident disease in the lateral neck (e.g. lymphadenopathy on examination and suggestive changes to the lateral nodes on imaging) formed the basis for the LNDs on patients in the low-calcitonin group. One patient in this group exhibited a concurrent borderline preoperative calcitonin level of 195ng/l.

Preoperatively, seven patients in the high-calcitonin group, and four in the low had nodal involvement on imaging (cN1). Of those who were node-negative preoperatively (cN0), nine had histological nodal metastases (pN1). Eight were from the high-calcitonin group, with central nodes involved in only five patients. Two patients had distant metastases (M1) before starting adjuvant therapy.

Nine (36%) patients received adjuvant radiotherapy, one (4%) chemotherapy, one (4%) palliative chemotherapy and radiotherapy, 13 (52%) no therapy and one (4%) had none mentioned. Four patients (15%), all from the high-calcitonin group, had locoregional recurrence, of whom three (75%) underwent a central dissection and one (25%) a LND. Additionally, none received adjuvant therapy, and two (50%) had a RET mutation.

ENT consultants operated on 18 (72%) patients and general surgeons on seven (28%). No (0%) surgeries were complicated by chyle leak/bleeding. Five (20%) suffered nerve injuries—three (12%) in the high-calcitonin group and two (8%) in the low-calcitonin group. Four of the five (80%) who suffered nerve injuries had a LND. Two patients suffered recurrent laryngeal nerve injuries, two had injuries to cervical plexus branches, and one had an injury to both.

**Figure 1:**
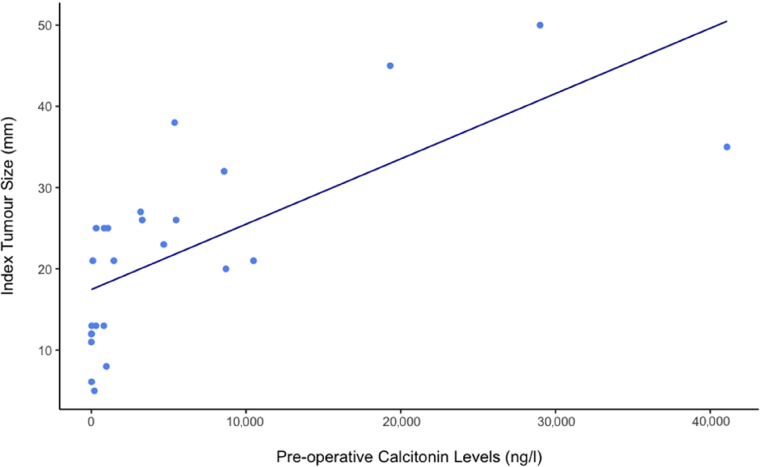
Scatter plot of preoperative calcitonin levels (ng/l) in thousands versus size of index tumor (mm). Pearson correlation coefficient found a moderate positive correlation between preoperative calcitonin levels and index tumour size (*R*=0.70, *p*<0.01).

A Pearson Correlation Coefficient found a significant, moderate positive correlation between preoperative calcitonin levels and index tumour size (*R*=0.70, *p*<0.01) (Figure 1).

**Figure 2:**
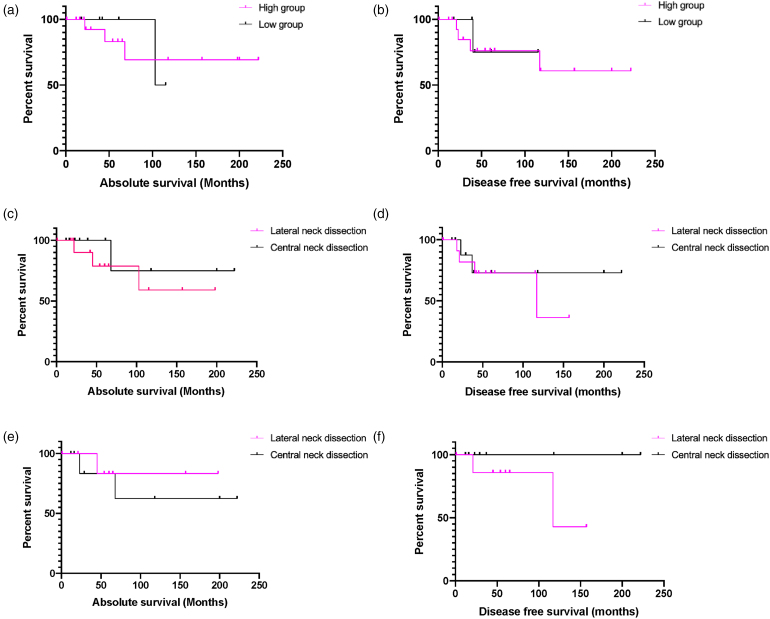
Kaplan–Meier curves. (a) Overall survival in patients with MTC stratified by preoperative calcitonin levels (high group *n*=18, low group *n*=7); log-rank *p*=0.960, HR 1.06 (95% CI 0.11–9.89). (b) Disease-free survival in patients with MTC stratified by preoperative calcitonin levels (high group *n*=18, low group *n*=7); log-rank *p*=0.817, HR 1.28 (95% CI 0.17–9.90). (c) Absolute survival in patients with MTC stratified by the type of neck dissection performed (LND *n*=14, CND *n*=11); log-rank *p*=0.444, HR 2.35 (95% CI 0.33–16.94). (d) Absolute survival in patients with MTC stratified by the type of neck dissection performed (LND *n*=14, CND *n*=11); log-rank *p*=0.591, HR 1.58 (95% CI 0.31–7.90). (e) Absolute survival in patients with MTC and a preoperative calcitonin >200ng/l (high-calcitonin group) stratified by the type of neck dissection performed (LND *n*=10, CND *n*=8); log-rank *p*=0.607, HR 0.55 (95% CI 0.06–5.30). (f) Disease-free survival in patients with MTC and a preoperative calcitonin >200ng/l (high-calcitonin group) stratified by the type of neck dissection performed (LND *n*=10, CND *n*=8); log-rank *p*=0.129, HR 8.784 (95% CI 0.53–145.1). CI = confidence interval; CND = central neck dissection; HR = hazard ratio; LND = lateral neck dissection; MTC = medullary thyroid carcinoma.

**Figure 3:**
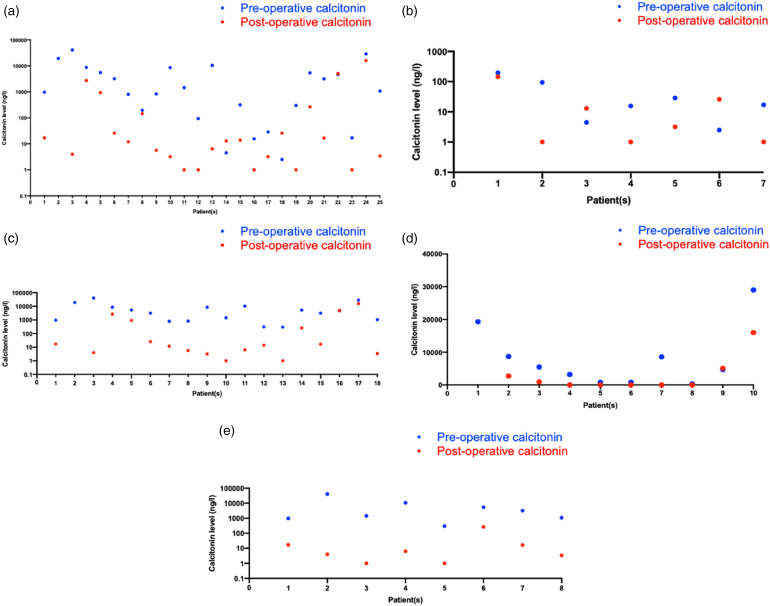
Graphs showing comparison between preoperative (blue) and most recent postoperative (red) calcitonin levels. (a) All patients (*n*=25). (b) Low-calcitonin group (*n*=7). (c) High-calcitonin group (*n*=18). (d) Patients in the high-calcitonin group undergoing LND (*n*=10). (e) Patients in the high-calcitonin group undergoing CND (*n*=8). CND = central neck dissection; LND = lateral neck dissection.

### Nodal yield

Across all patients, 994 lymph nodes were removed. A total of 256 (26%) were cancerous based on histopathology. In the high-calcitonin group, 817 nodes were removed, with 230 (28%) being cancerous. In the low-calcitonin group, 177 nodes were removed, with 26 (15%) being histologically involved.

An independent *t*-test showed no difference between the number of nodes removed (mean difference=19.1, *p*=0.24), nodes involved (mean difference=8.7, *p*=0.056), and proportion of involved nodes (mean difference=9.7%, *p*=0.41) between the two groups. Table 2 shows the nodal yield and involvement at each level.

**Table 2:** Number of nodes removed, involved and proportion (%) at each level in the ipsilateral and contralateral neck

Levels	High (*N*=18)			Low (*N*=7)			Total (*N*=25)		
	Involved	Removed	%	Involved	Removed	%	Involved	Removed	%
Ipsilateral I	2	15	13	0	0	–	2	15	13
Ipsilateral II	46	185	25	1	52	2	47	237	20
Ipsilateral III	41	80	51	8	28	29	49	108	45
Ipsilateral IV	28	83	34	2	26	8	30	109	28
Ipsilateral V	24	85	28	1	27	4	25	112	22
Contralateral I	0	0	–	0	0	–	0	0	–
Contralateral II	6	67	9	0	0	–	6	67	9
Contralateral III	3	63	5	0	0	–	3	63	5
Contralateral IV	9	65	14	0	0	–	9	65	14
Contralateral V	0	24	0	0	0	–	0	24	0
Level VI (Central)	71	149	48	14	44	32	85	193	44%
Level VII (Mediastinal)	0	1	0	0	0	–	0	1	0%

### Survival analysis

Survival analysis using log-rank (Mantel–Cox) testing found no significant difference in absolute survival (*p*=0.960, hazard ratio (HR) 1.06, 95% confidence interval (CI) 0.11–9.89) or disease-free survival (*p*=0.817, HR 1.28, 95% CI 0.17–9.90) between the high and low-calcitonin groups (Figure 2a, b).

There was also no significant difference in absolute or disease-free survival between all patients who had LNDs and CNDs across both groups. Interestingly, and statistically insignificant, the HR was over two times higher in the LND group with respect to absolute survival (HR 2.35, 95% CI 0.33–16.94, *p*=0.444) (Figure 2c, d).

Subgroup analysis of central vs lateral neck dissection in the high-calcitonin group found no significant difference in absolute survival (*p*=0.607, HR 0.55, 95% CI 0.06–5.30) or disease-free survival (*p*=0.129, HR 8.78, 95% CI 0.53–145.1) (Figure 2e, f).

### Preoperative vs postoperative calcitonin

Of all 25 patients in the study, only 3 (12%) saw a rise from their preoperative calcitonin to their most recent postoperative level. Of these, two were in the low-calcitonin group and had CND, and one was in the high-calcitonin group and had LND (Figure 3a–c).

The patient in the high-calcitonin group with a higher postoperative calcitonin level had identified mediastinal metastases (M1 disease) and received no adjuvant therapy. The two patients in the low-calcitonin group with higher calcitonin postoperatively had no identifiable disease recurrence and received no adjuvant therapies. One patient in the high-calcitonin group had no recorded postoperative calcitonin.

Nine of the ten (90%) patients in the high-calcitonin group who underwent LND had a lower postoperative calcitonin compared with their preoperative level (Figure 3d). Similarly, all patients in the high-calcitonin group who underwent CND saw their most recent postoperative calcitonin level fall below their preoperative level (Figure 3e).

## Discussion

This study assessed oncological outcomes for patients with MTC from a single institution, focusing on the use of preoperative calcitonin levels and subsequent lateral/central neck dissection. A preoperative calcitonin cut-off of 200ng/l was used to delineate high- from low-calcitonin groups.^10,15^

Overall, there was no statistically significant difference in overall survival or disease-free survival between groups. In each group, the extent of neck dissection for each patient was guided by MDT consensus, as opposed to randomisation. The low-calcitonin group was, on average, 13.4 years older. Age, postoperative calcitonin, index tumour size and nodal involvement have all previously been found to impact survival.^16,17^

Poorer survival associated with age described in existing literature has the potential to bias survival outcomes in the low-calcitonin group towards the poorer end of the spectrum—something not seen with our data.

### Cutoff value

As previously referenced, the use of a cutoff value of 200ng/l to determine high- and low- calcitonin groups was based on study findings by Machens *et al*.^10^ Patients with a preoperative calcitonin >50ng/l had an increased likelihood of ipsilateral lateral neck metastases. Those with a calcitonin >200ng/l had an increased likelihood of contralateral neck metastases. Given the findings of Machens *et al*, the use of a lower cutoff value (e.g. 100ng/l) would likely decrease the proportion of those in the high-calcitonin group who had lateral neck metastases. Conversely, a higher cutoff value would increase the proportion of patients in the high-calcitonin group who had lateral neck metastases.

### Index tumour size

The average index tumour size was 10.7mm larger in the high-calcitonin group. Index tumours larger than 20mm have been associated with increased disease-specific mortality.^17^ Higher calcitonin levels have been correlated previously with larger index tumours and more nodal metastases.^10^ The Pearson correlation coefficient showed a statistically significant positive correlation between the index tumour size and the preoperative calcitonin level (*R*=0.70; *p*<0.01). Altogether, this could suggest that preoperative calcitonin >200ng/ml should be used as an indicator of the need for a prophylactic LND.

We found that calcitonin was a good diagnostic marker for MTC and was correlated with tumour size. However, it was inadequate for identifying nodal metastases, as there was no significant difference in the number or proportion of involved nodes between the high and low groups. Previous studies suggest that prophylactic LND in patients with a calcitonin >200ng/l is not associated with improved survival outcomes.^18^

### Preoperative vs postoperative nodal status

Eight patients from the high-calcitonin group who were node-negative preoperatively (cN0) had nodal involvement on postoperative histology (pN1). The affected nodes were from the central neck only in five of these eight (63%). This suggests that if patients are initially node negative (cN0), despite high calcitonin levels, they only had nodal involvement in the central neck, so were still node negative in the lateral neck. Therefore, there may be benefit to performing selective LND as opposed to LND based on a high-calcitonin level alone.

### Survival data

At 60 months, the survival probability in the high-calcitonin group was 83% compared with 100% in the low-calcitonin group. This could be a potentially clinically significant difference, albeit not statistically significant due to MTC being a rare disease with a small patient cohort.

Given patients with a preoperative calcitonin >200ng/l should ideally receive a prophylactic LND, records were reviewed to ascertain why eight in the high-calcitonin group underwent a CND only. Comorbidities and MDT decisions surrounding lack of clinically evident disease in the lateral neck formed the basis for this.

Subgroup analysis of the high-calcitonin group, albeit statistically insignificant, showed that there was a difference in absolute survival at 100 months between those who had a LND (83% survival probability) and those who had a CND (63% survival probability). The lack of significance could be viewed as a reason to use a selective approach to LND, even in the presence of a calcitonin >200ng/l, due to the increased complications associated with LND and lack of a significantly improved survival.^19^

Furthermore, the disease-free survival probability at 100 months was lower in those that had a LND (86% vs 100%). The difference was even more pronounced at 150 months (43% vs 100%). Despite the small sample size, this suggests that the intended benefit of a prophylactic LND (early removal of micrometastases to prevent disease spread/recurrence) is not seen long term. This raises the question of whether a selective approach to LND based on clinical and radiological evidence would be better, given the higher risk of complications associated with the procedure.

Young patients with familial disease are at high risk of locoregional recurrence^20^; therefore, this group would potentially benefit from a more aggressive surgical approach.

### Strengths and limitations

This study has several limitations. First, retrospective studies come with inherent bias. Additionally, significant advances in imaging modalities and systemic treatments during the study period create the potential for errors generated by comparing eras. Finally, as this is a single-centre study, associations and outcomes were assessed on 25 patients only, underpowering the findings.

Conversely, acknowledging that MTC is rare (only 1–2% of all thyroid cancers) is important.^2^ A study reflecting on a single centre's experiences, like ours, may lack statistical power but is hypothesis generating. Spanheimer *et al* demonstrated similar findings with a much larger study (316 patients) over a longer timeframe (31 years vs 21 years in our study).^18^ Furthermore, this study creates scope for a larger, multicentre study across London, or other national centres, in the near future for validation.

## Conclusion

In conclusion, we found no statistically significant difference in oncological outcomes between high- and low-calcitonin groups. Suggestions that calcitonin levels above 200 ng/l should be used to guide need for a prophylactic LND are therefore debatable without a survival advantage. The alternative of performing selective LND based on advanced imaging, such as Dotatate positron emission tomography-CT scanning, should be explored further, given the complication rate of a LND increases as the LND range becomes more radical. Accordingly, ensuring lateral nodal involvement before undertaking a LND is of great importance in decreasing the incidence of major complications.

## References

[1] Wong A, Nabata K, Wiseman SM. Medullary thyroid carcinoma: a narrative historical review. *Expert Rev Anticancer Ther* 2022; **22**: 823–834. 35694971 10.1080/14737140.2022.2089118

[2] Kushchayev SV, Kushchayeva YS, Tella SH *et al.* Medullary thyroid carcinoma: an update on imaging. *J Thyroid Res* 2019; **2019**: 1893047. 31360432 10.1155/2019/1893047 PMC6642782

[3] Roman S, Lin R, Sosa JA. Prognosis of medullary thyroid carcinoma. Demographic, clinical, and pathologic predictors of survival in 1252 cases. *Cancer* 2006; **107**: 2134–2142. 17019736 10.1002/cncr.22244

[4] Thomas CM, Asa SL, Ezzat S *et al.* Diagnosis and pathologic characteristics of medullary thyroid carcinoma-review of current guidelines. *Curr Oncol* 2019; **26**: 338–344. 31708652 10.3747/co.26.5539 PMC6821118

[5] Perros P, Boelaert K, Colley S *et al.* Guidelines for the management of thyroid cancer. *Clin Endocrinol (Oxf)* 2014; **81**: 1–122. 10.1111/cen.12515 24989897

[6] Engelbach M, Görges R, Forst T *et al.* Improved diagnostic methods in the follow-up of medullary thyroid carcinoma by highly specific calcitonin measurements. *J Clin Endocrinol Metab* 2000; **85**: 1890–1894. 10843170 10.1210/jcem.85.5.6601

[7] Cohen R, Campos JM, Salaün C *et al.* Preoperative calcitonin levels are predictive of tumor size and postoperative calcitonin normalization in medullary thyroid carcinoma. groupe d’Etudes des tumeurs a calcitonine (GETC). *J Clin Endocrinol Metab* 2000; **85**: 919–922. 10690910 10.1210/jcem.85.2.6556

[8] Machens A, Schneyer U, Holzhausen H *et al.* Prospects of remission in medullary thyroid carcinoma according to basal calcitonin level. *J Clin Endocrinol Metab* 2005; **90**: 2029–2034. 15634717 10.1210/jc.2004-1836

[9] Machens A, Lorenz K, Dralle H. Individualization of lymph node dissection in RET (rearranged during transfection) carriers at risk for medullary thyroid cancer: value of pretherapeutic calcitonin levels. *Ann Surg* 2009; **250**: 305–310. 19638924 10.1097/SLA.0b013e3181ae333f

[10] Machens A, Dralle H. Biomarker-based risk stratification for previously untreated medullary thyroid cancer. *J Clin Endocrinol Metab* 2010; **95**: 2655–2663. 20339026 10.1210/jc.2009-2368

[11] Ahmed SR, Ball DW. Clinical review: incidentally discovered medullary thyroid cancer: diagnostic strategies and treatment. *J Clin Endocrinol Metab* 2011; **96**: 1237–1245. 21346073 10.1210/jc.2010-2359 PMC3085196

[12] Chambon G, Alovisetti C, Idoux-Louche C *et al.* The use of preoperative routine measurement of basal serum thyrocalcitonin in candidates for thyroidectomy due to nodular thyroid disorders: results from 2733 consecutive patients. *J Clin Endocrinol Metab* 2011; **96**: 75–81. 20881258 10.1210/jc.2010-0162

[13] Jin LX, Moley JF. Surgery for lymph node metastases of medullary thyroid carcinoma: a review. *Cancer* 2016; **122**: 358–366. 26539937 10.1002/cncr.29761

[14] Machens A, Gimm O, Ukkat J *et al.* Improved prediction of calcitonin normalization in medullary thyroid carcinoma patients by quantitative lymph node analysis. *Cancer* 2000; **88**: 1909–1915. 10760769

[15] Wells SA, Asa SL, Dralle H *et al.* Revised American thyroid association guidelines for the management of medullary thyroid carcinoma. *Thyroid* 2015; **25**: 567–610. 25810047 10.1089/thy.2014.0335 PMC4490627

[16] Ho AS, Wang L, Palmer FL *et al.* Postoperative nomogram for predicting cancer-specific mortality in medullary thyroid cancer. *Ann Surg Oncol* 2015; **22**: 2700–2706. 25366585 10.1245/s10434-014-4208-2 PMC4986610

[17] Kuo EJ, Sho S, Li N *et al.* Risk factors associated with reoperation and disease-specific mortality in patients with medullary thyroid carcinoma. *JAMA Surg* 2018; **153**: 52–59. 28973144 10.1001/jamasurg.2017.3555 PMC5833622

[18] Spanheimer PM, Ganly I, Chou JF *et al.* Prophylactic lateral neck dissection for medullary thyroid carcinoma is not associated with improved survival. *Ann Surg Oncol* 2021; **28**: 6572–6579. 33748897 10.1245/s10434-021-09683-8 PMC8452790

[19] Sakorafas GH, Sampanis D, Safioleas M. Cervical lymph node dissection in papillary thyroid cancer: current trends, persisting controversies, and unclarified uncertainties. *Surg Oncol* 2010; **19**: e57–e70. 19447608 10.1016/j.suronc.2009.04.002

[20] Spanheimer PM, Ganly I, Chou J *et al.* Long-term oncologic outcomes after curative resection of familial medullary thyroid carcinoma. *Ann Surg Oncol* 2019; **26**: 4423–4429. 31549322 10.1245/s10434-019-07869-9 PMC6876629

